# Exploring the Patterns of Gallbladder Diseases and Associated Risk Factors in a Tertiary Care Hospital of Mardan, Khyber Pakhtunkhwa Province, Pakistan

**DOI:** 10.7759/cureus.78093

**Published:** 2025-01-27

**Authors:** Sumaira Noureen, Niamat Ali Khan, Ambreen Shahid, Muhammad Idrees, Mansoor Ahmed, Muhammad Haseeb Shah, Hamza Khan, Hajra Faheem, Mah Noor Shaukat, Sahar Zahoor

**Affiliations:** 1 Department of Radiology, Mardan Medical Complex, Mardan, PAK; 2 Department of Radiology, Mardan medical complex, Mardan, PAK; 3 Department of General Surgery, Mardan Medical Complex, Mardan, PAK; 4 Department of Pediatrics, Mardan Medical Complex, Mardan, PAK; 5 Department of Internal Medicine, Northwest General Hospital and Research Center, Mardan, PAK; 6 Department of Public Health, Khyber Medical University, Mardan, PAK

**Keywords:** cholecystectomy, cholecystitis, cholelithiasis, gallbladder complications, gallbladder diseases

## Abstract

Background

Gallbladder diseases represent a significant contributor to global morbidity. The most common pathological conditions affecting the gallbladder include cholelithiasis, calculous and acalculous cholecystitis, gallbladder gangrene, and perforation. Gallbladder diseases are a common health concern in European and American societies, with a significant number of new cases diagnosed annually and a substantial number of surgical procedures performed each year to address the condition. This study aims to specify complications and the predisposing risk factors affecting mortality and morbidity in patients with gallbladder diseases who underwent surgery.

Patients and methods

We analyzed medical records of 42 patients who were diagnosed with gallbladder diseases and had a cholecystectomy (open+laparoscopic) in a span of one year between August 2023 and August 2024. Statistical calculations were performed using the SPSS version 26 (IBM Corp., Armonk, NY, USA).

Results

Data from 42 patients who underwent cholecystectomy (open+laparoscopic) for gall bladder diseases over a period of one year were collected and analyzed. This included 24 male and 18 female patients between 24 and 85 years old with a mean age of 58.74 ± 16.049. Pain abdomen (n=40, 95.2%) was the most common symptom in all the study subjects followed by nausea, vomiting (n=33, 78.6%), and fever (n=26, 61.9%). The majority of the patients were diagnosed with cholecystitis(calculus+acalculus) followed by gangrenous perforated gallbladder, empyema of the gallbladder, and gallbladder sludge. The occurrence of comorbidities like hypertension, diabetes mellitus, and ischemic heart disease was very high in the age group 61-70 years with a male predominance.

Conclusion

In conclusion, the results of this study suggest that cholecystitis (calculus and acalculus) is the more commonly occurring gallbladder disease. The possible predisposing factors are old age, raised white blood cell count, and comorbid conditions like hypertension, diabetes mellitus and ischemic heart disease which probably are the main cause of gallbladder disease in our population with a male predominance.

## Introduction

The gallbladder is a pear-shaped organ located on the inferior surface of the liver near the confluence of the right and left lobes and serves as a bile reserve [1,2]. Cholelithiasis, calculus, and acalculous cholecystitis are three common pathological conditions linked to the gallbladder that place a significant financial burden on the healthcare system even in Western nations where it is estimated that 10% to 20% of people in European and American societies experience gallstones [1,2]. Gallbladder illnesses are a major cause of global morbidity, with varied degrees of frequency depending on geographical, racial, and ethnic factors [3,4]. Every year, around one million new patients are diagnosed in the United States, and roughly 600,000 cholecystectomy operations are performed [3,4]. Gallstone prevalence in Pakistan has been estimated at 10% [5]. In the majority of cases, cholecystectomy, one of the most popular abdominal surgical operations worldwide, is performed with little to no risk of serious consequences, however bile leakage and intraoperative contamination may lead to surgical site infection [5].

The need for cholecystectomy in individuals with gallbladder stones is usually determined by the presenting symptoms, such as indigestion or intermittent stomach discomfort, which do not require surgical intervention. However, if the patient has recurring biliary colic attacks or has a severe form of gallbladder inflammation such as acute cholecystitis, then it requires urgent intervention [6-8]. To identify the risk factors in a given population, it is essential that epidemiological studies first define the frequency of disease [7]. Acute cholecystitis can be considered the most common complication of biliary stones [8]. The severe type is called gangrenous or necrotizing cholecystitis which requires emergency cholecystectomy. In other words, gangrenous cholecystitis is a type of acute cholecystitis with a combination of transmural inflammation, mucosal abscess, and necrosis of the gallbladder wall, the incidence of this complication with acute cholecystitis is between 2% and 30%, with high mortality and high morbidity rate (0.2-0.9%) [9-11]. Various studies on gangrenous cholecystitis have indicated that male gender, increasing age, cardiovascular disease, and diabetes are risk factors for the development of gangrenous cholecystitis [11].

As per the literature, it has been found that secondary acute cholecystitis develops in 1% to 2% of patients with gallstones; amongst this, about 2% to 11% of people affected further progress towards gallbladder perforation. Gallbladder perforation is classified according to Niemeier classification. Although gallbladder perforation is rare its incidence does make it a life-threatening complication having a mortality rate of about 12-42% [12-14]. The hypothesized epidemiologic triggers for gallbladder disease in adulthood are female gender, race, and obesity, In the adult population, the correlation between obesity and gallbladder ailments is widely known [15].

Our study aims to specify complications and the predisposing risk factors affecting mortality and morbidity in patients with gallbladder diseases who underwent surgery.

## Materials and methods

Study design

This study utilized a cross-sectional observational design, focusing on collecting and analyzing data at a specific point in time to identify patterns and associations within the target population.

Study population

We analyzed the medical records of 42 patients who were diagnosed with gallbladder diseases and had a cholecystectomy (open+laparoscopic) in a span of one year between August 2023 and August 2024.

Sample selection

Subjects with significant co-morbidities such as chronic obstructive pulmonary disease (COPD), stroke, HIV, chronic hepatitis B and C, acute pancreatitis, or abnormal blood coagulation were all excluded. 

Before data collection, we selected several factors that we postulated might increase morbidity and mortality rate. Among these were diabetes, hypertension, and cardiovascular and renal diseases.

In addition to demographic data (age, sex, PR #), underlying chronic illnesses (diabetes mellitus, coronary artery disease, hyperlipidemia), positive history, and preoperative physical examination (pain or mass or tenderness in right upper quadrant (RUQ), nausea, vomiting, fever), and early laboratory findings (white blood cell count, blood urea nitrogen (BUN), creatinine, aspartate aminotransferase (AST), alanine aminotransferase (ALT), albumin, alkaline phosphatase, bilirubin, amylase, lipase), and preoperative imaging (ultrasound) were included in the study.

Data analysis

Statistical calculations were performed using the SPSS version 26 (IBM Corp., Armonk, NY, USA). Mean, standard deviation, frequencies and percentages were calculated for continuous as well as categorical variables. The findings are presented in graphical and tabular formats. The Chi-square test was performed. P-values of ≤0.05 were considered significant.

Study measures

The study included patients aged 24 to 85 years, with a mean age ranging across this spectrum. The sex distribution was 24 males and 18 females. The most common symptoms presented were abdominal pain (95.2%), nausea and vomiting (78.6%), and fever (61.9%). Diagnoses made included cholecystitis (both calculous and acalculous), gangrenous or perforated gallbladder, empyema of the gallbladder, and gallbladder sludge. Surgical interventions performed were open cholecystectomy and laparoscopic cholecystectomy.

Ethical consideration

All the data collected is treated with utmost confidentiality, and no personally identifiable information is disclosed in any publication or presentation.

## Results

Age and gender

**Figure 1:**
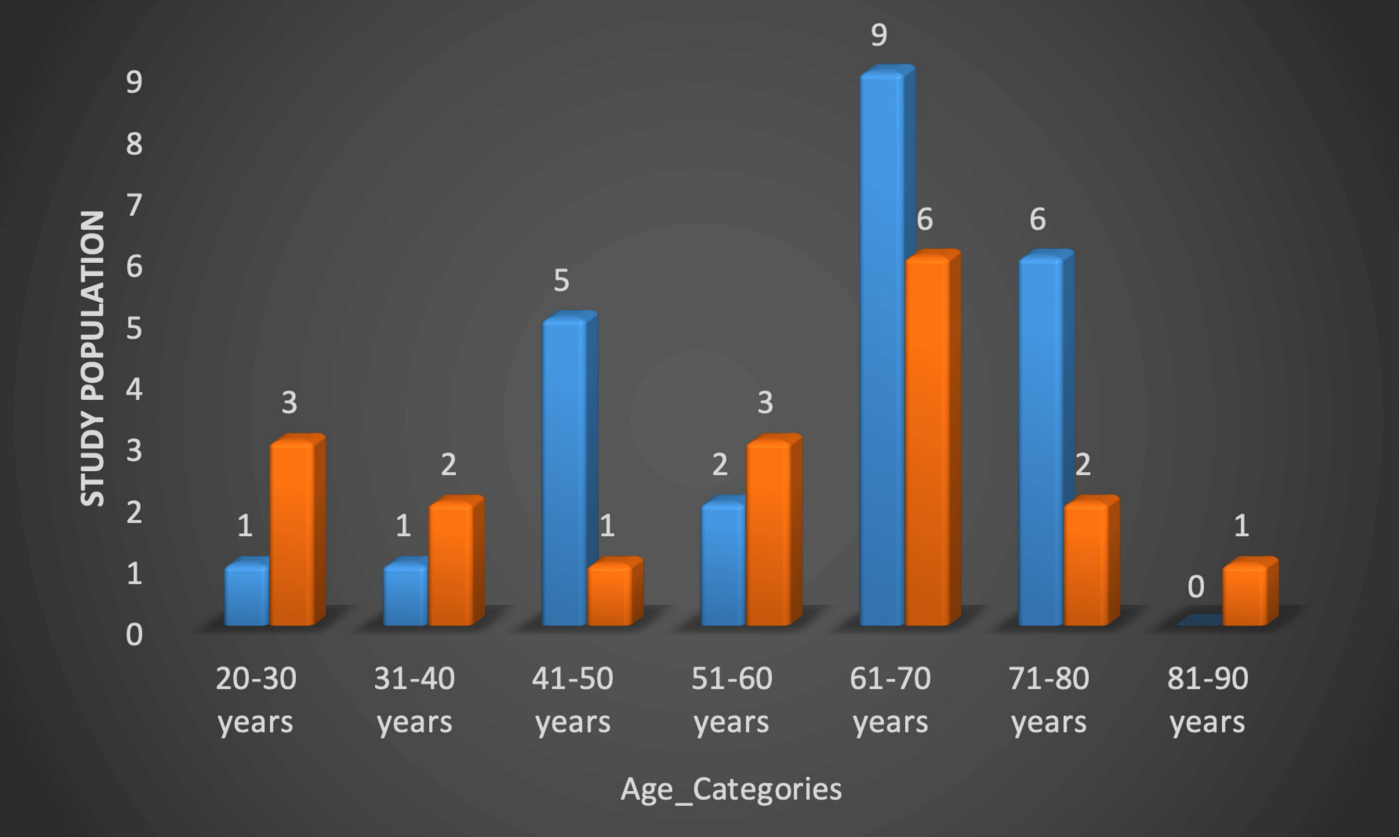
Age and sex distribution of the study population.

Data from 42 patients who underwent cholecystectomy (open+laparoscopic) for gall bladder diseases over one year were collected and analyzed. This included 24 males and 18 female patients who were between 24 and 85 years old with a mean age of 58.74 ± 16.049. Most of the patients (62%) presented between the age group 41-70 years and 21.4% were in the age group of >70 years. Males (n=24) were more frequently affected than females (n=18) especially in the age group of 61-70 years with a male to female ratio of 4:3 (Figure 1).

Clinical presentation

With regard to clinical presentation, the most prevalent symptom in all study individuals was abdominal discomfort (n=40, 95.2%), followed by nausea, vomiting (n=33, 78.6%), and fever (n=26, 61.9%). Only two patients had complained of loose motion (4.8%). Approximately 65% of the patients had three or more symptoms overall (Table 1).

**Table 1:** Patients' demographic and characteristic findings

Age-Categories	Frequency (N)	Percent (%)
20-30 years	4	9.5
31-40 years	3	7.1
41-50 years	6	14.3
51-60 years	5	11.9
61-70 years	15	35.7
71-80 years	8	19.0
81-90 years	1	2.4
Gender		
Male	24	57.1
Female	18	42.9
Presenting Complaint		
Abdominal pain	40	95.2
Nausea, Vomiting	33	78.6
Loose motion	2	4.8
Fever	26	61.9
Lab Findings		
WBC		
Less than 4000	1	2.5
4000 to 15000	25	59.5
More than 15000	12	31.6
Diagnosis		
Calculus Cholecystitis	07	16.7
Acalculus Cholecystitis	17	40.5
Gallbladder Sludge	03	7.1
Gangrenous perforated	07	16.7
Empyema Gallbladder	04	9.5
Gallbladder polyp	01	2.4
Disseminated malignancy	03	7.1

Diagnosis

54.8% of patients were diagnosed with acute cholecystitis(calculus+acalculus), followed by gangrenous perforated gallbladder (16.7%), empyema of gallbladder (9.5%) gallbladder sludge (7.1%) disseminated malignancy (7.1%) and gallbladder polyp (2.4%) (Table 1).

Comorbidities

**Figure 2:**
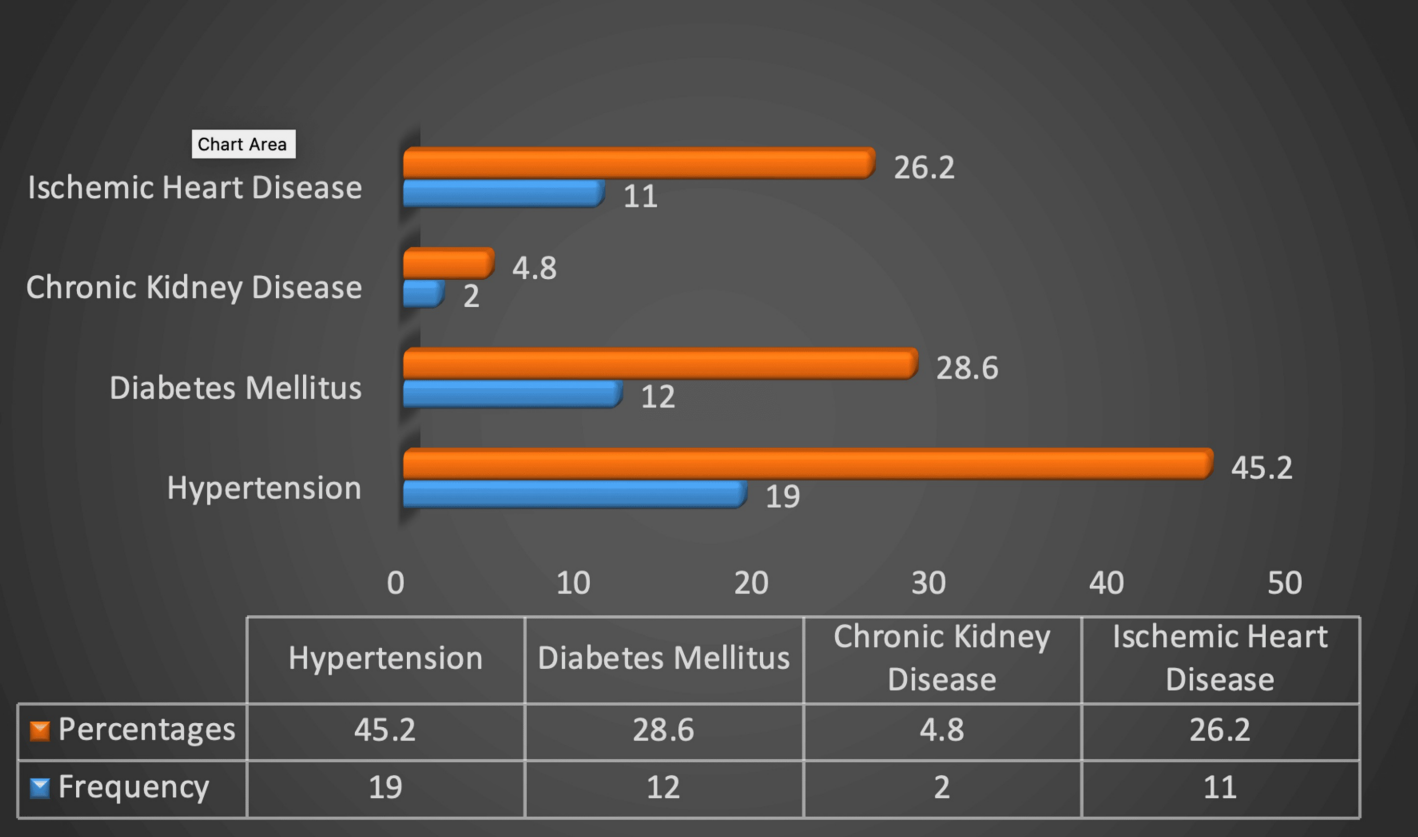
Frequency and percentages of comorbidities in patients with gallbladder disease (p>0.05).

The incidence of comorbidities such as hypertension, diabetes mellitus, and ischemic heart disease was particularly common in people aged 61 to 70 years, with a male predominance and significance of 0.000, 0.001 and 0.002 respectively (Figure 2).

Niemeier classification

A total of 15 cases of gallbladder perforation were found to be co-existing with cholelithiasis. In our study Type I gallbladder perforation (n=13, 86.7%) (Niemeier classification, Table 2) was found to be more common followed by Type II gallbladder perforation (n=2, 13.3%). This investigation found no type III GBP (Table 3).

**Table 2:** Niemeier’s classification for gallbladder perforation. Author Credits: Sumaira Noureen

Type	Onset	Explanation
Type1	Acute	Involved the perforation of the gallbladder into the peritoneal cavity; it is not surrounded by any protective adhesions
Type2	Subacute	Consists of a perforated gallbladder surrounded by an abscess that is walled off by adhesions
Type3	Chronic	Includes the formation of a fistula between the gallbladder and some other viscera

**Table 3:** Frequency and valid percentage of different types of gall bladder perforation.

Type of Perforation	Frequency	Valid Percentage
TYPE 1	13	86.7
TYPE 2	02	13.3
TYPE 3	0	0

CT scan and ultrasound findings

Ultrasound and CT scan findings are presented in Table 4 and Table 5 respectively. All the patients were discharged with no post-operative complications.

**Table 4:** Ultrasound Findings

Ultrasound Findings	Frequency & (Percent)	P- Value
Wall_thickening_and_edema	11 (27.5)	0.329
Pericholecystic_fluid_collection	6 (15)	0.254
Gallblader_with_sludge_and_calculi	24 (60.0)	0.010

**Table 5:** CT scan Findings

CT Scan Findings	Frequency & (percentage)	P-Value
Wall_thickening_and_edema	15 (37.5)	0.123
Irregular_or_absent_gallbladder_wall	10 (25)	0.459
Empyema	02 (5)	0.004

## Discussion

The majority of our patients had several ailments, like hypertension, diabetes mellitus and ischemic heart disease that probably predisposed them to gallbladder diseases. It is crucial to provide an accurate and timely diagnosis because severe complications would be reduced [9]. Forty-two patients who were presented to our setting with gallbladder diseases (who underwent cholecystectomy, open or laparoscopic) were analyzed over a period of one year. That included 24 male and 18 female patients with an age range between 24 and 85 years with a mean age of 58.74 ± 16.049. Most of the patients (62%) presented between the age group 41-70 years. This study shows males (n=24) were more frequently affected than females (n=18) especially in the age group of 61-70 years with a male-to-female ratio of 4:3 (Figure 1). Gallbladder diseases are generally more prevalent in older people in contrast to younger ones. In this study, the most commonly affected age group was 61-70 years. Similarly, studies done in Mackay Memorial Hospital, Taiwan, China, and Mysore Medical College, Karnataka, India also suggested that the older age group is more frequently affected than the age groups of 50-60 years and 61-65 years respectively [2,9]. In this study, gallbladder diseases were more common in males as compared to females with a percentage of 57.1%. our results agreed with the previously published studies at Sheikh Khalifa Bin Zayed Al Nahyan (SKBZ) and CMH Hospital Muzaffarabad and Balıkesir University, Turkey [10,11]. Males made up a higher proportion of patients in our sample (57.1%, n=24), indicating the tendency towards gender bias.

A systematic literature analysis has called into doubt the predictive utility of clinical symptoms or laboratory tests in the diagnosis of acute cholecystitis [12]. Parker et al. stated that fever, right upper quadrant discomfort, and leukocytosis have been reported as common but vague symptoms [13]. Our study, on the other hand, reported that individuals with advanced age, a high white blood cell count, and certain conditions such as hypertension, diabetes mellitus, and ischemic heart disease were related to a greater prevalence of gallbladder disease [9,11].

At admission, all patients had abdominal ultrasounds which revealed gallbladder wall anomalies. Ultrasound was helpful in determining the need for surgical intervention [11,14]. CT scan, on the other hand, appears to increase diagnosis accuracy. Thin slices of CT can also reveal gallbladder wall thickness and irregular or missing gallbladder wall [15].

In this study cholecystitis (acute and chronic) was the most common diagnosis of all gallbladder diseases with a percentage of 54.8% followed by gangrenous perforated gallbladder (19.1%), empyema of the gallbladder (9.5%) gallbladder sludge (7.1%), disseminated malignancy (7.1%) and gallbladder polyp (2.4%). In comparison with a study done in the Department of Surgery, Nalanda Medical College, Patna also suggested that cholecystitis (acute and chronic) with a percentage of 57.1% is most common with male predominance [16]. 

Gallbladder perforation is a rare consequence of acute cholecystitis that affects about 3% of patients and is frequently accompanied by the presence of stones. The author came across 15 cases of gallbladder perforation during the study period with ages ranging from 41 to 80 years. The most frequently afflicted age range was 61 to 70. There were eight female and seven male cases out of 15. This observation is in agreement with a study conducted in Mysore Medical College, India where the most prevalent age range impacted was 61-65 years, and out of 12 instances, seven were females and five were men [2].

This study found that cholecystectomized patients had a 35.7% incidence of GBP, with the diagnosis of GBP based on surgical findings. The prevalence of type I GBP was higher (86.7%) than type II GBP (13.7%). During the time of the study, the author did not encounter any type III GBP.

The mortality rate ranges from 12 to 42% [17,18]. Gallbladder perforation was categorised by Niemeier as acute or type I generalised peritonitis, subacute or type II pericholecystic abscess and localised peritonitis, and chronic or type III cholecystoenteric fistula (Table 2) [19].

Cholecystectomy, which was formerly performed via open laparotomy, is the basis of therapy for gallbladder ailments. But In a recent modest trial, laparoscopic cholecystectomy was carried out without any complications.

Limitations

The limitations of this study include a relatively small sample size, which may affect the generalizability of the findings, and the fact that all data were obtained from a single medical center, potentially limiting the applicability of the results to broader populations or settings.

## Conclusions

In conclusion, the results of this study suggest that cholecystitis(calculus and acalculus) is the more commonly occurring gallbladder disease followed by gangrenous perforated gallbladder and empyema. Furthermore, the possible predisposing factors that are different in our study include old age and comorbid conditions like hypertension, diabetes mellitus and ischemic heart disease which probably are the main cause of gallbladder disease in our population with a male predominance. Early and accurate diagnosis is essential because severe complications would be reduced. besides clinical presentation, sonological investigation also helps in determining the need for surgical intervention. Our results demonstrate as well that the preferable therapy for gallbladder disease in our population is cholecystectomy. Also, the likelihood of problems from gallbladder illnesses may be reduced with better knowledge and early screening by healthcare professionals.
